# Mutation screening of the *CYP1B1* gene reveals thirteen novel disease-causing variants in consanguineous Pakistani families causing primary congenital glaucoma

**DOI:** 10.1371/journal.pone.0274335

**Published:** 2022-09-09

**Authors:** Raeesa Tehreem, Anam Arooj, Sorath Noorani Siddiqui, Shagufta Naz, Kiran Afshan, Sabika Firasat

**Affiliations:** 1 Department of Zoology, Faculty of Biological Sciences, Quaid-i-Azam University, Islamabad, Pakistan; 2 Department of Pediatric Ophthalmology and Strabismus, Al-Shifa Trust Eye Hospital, Rawalpindi, Pakistan; 3 Department of Zoology, Lahore College for Women University, Lahore, Pakistan; Government College University Faisalabad, PAKISTAN

## Abstract

**Background:**

Primary congenital glaucoma (PCG) is a heterogeneous rare recessively inherited disorder prevalent in regions with high consanguinity. Disease phenotype is associated with increased intra ocular pressure and is a major cause of childhood blindness. Sequence variations in *Cytochrome P450 1B1* (*CYP1B1*) gene are a major cause of PCG. Current study was conducted to screen *CYP1B1* gene in highly consanguineous PCG affected families from Pakistani population consistent with the autosomal recessive pattern of PCG inheritance.

**Methods:**

For this study, patients and controls (clinically unaffected individuals of each family) from 25 consanguineous families belonging to Punjab, Baluchistan and Khyber Pakhtunkhwa, Pakistan were recruited through ophthalmologists. DNA was isolated from collected blood samples. Genetic screening of *CYP1B1* gene was done for all enrolled families. In-silico analysis was performed to identify and predict the potential disease-causing variations.

**Results:**

Pathogenicity screening revealed sequence variants segregating with disease phenotype in homozygous or compound heterozygous form in eleven out of 25 analyzed families. We identified a total of sixteen disease causing variants among which five frameshift i.e., c.629dup (p.Gly211Argfs*13), c.287dup (p.Leu97Alafs*127), c.662dup (p.Arg222Profs*2), c.758_759insA (p.Val254Glyfs*73) and c.789dup (p.Leu264Alafs*63), two silent c.1314G>A, c.771T>G and six missense variations c.457C>G (p.Arg153Gly), c.516C>A (p.Ser172Arg), c.722T>A (p.Val241Glu), c.740T>A (p.Leu247Gln), c.1263T>A (p.Phe421Leu), and c.724G>C (p.Asp242His) are previously un reported. However two frameshift c.868dup (p.Arg290Profs*37), c.247del (p.Asp83Thrfs*12) and one missense variant c.732G>A (p.Met244Ile), is previously reported. Furthermore, six polymorphisms c.1347T>C, c.2244_2245insT, c.355G>T, c.1294G>C, c.1358A>G and c.142C>G were also identified. In the intronic region, a novel silent polymorphism i.e., g.35710_35711insT was found in homozygous state. All the newly detected disease-causing variants were negative in 96 ethnically matched controls.

**Conclusion:**

Among twenty-five screened families, eight families (PCG50, 52–54, 58, 59, 63 and 67) were segregating disease causing variants in recessive manner. Two families (PCG049 and PCG062) had compound heterozygosity. Our data confirms genetic heterogeneity of PCG in Pakistani population however we did not find molecular variants segregating with PCG in fifteen families in coding exons and intron-exon boundaries of *CYP1B1* gene. Genetic counseling was provided to families to refrain from practicing consanguinity and perform premarital screening as a PCG control measure in upcoming generations.

## Introduction

Glaucoma is characterized by impaired vision due to increased intraocular pressure, a primary risk factor for irreversible optic nerve damage. This disorder can be categorized according to etiology (primary glaucoma/secondary glaucoma), onset (congenital/adult) and iridocorneal angle (open/close) [1]. Primary congenital glaucoma (PCG; OMIM 231300) manifests during the first three years of life due to developmental defects of trabecular meshwork and Schlemm’s canal resulting in hindrance to outflow of aqueous humour and increased intra-ocular pressure [2]. Clinical manifestation of PCG includes buphthalmos, epiphora, photophobia, hyperlacrimation, optic nerve damage, blepharospasm (uncontrolled eyelid movement), enlarged and opaque cornea [3]. Worldwide prevalence of PCG is 1:10,000 to 18,000 live births [4] with males (65%) being more affected than females (35%) [5] but there is variability of incidence between populations [1, 6]. Genetically PCG is heterogeneous with incomplete penetrance and four genetic loci are reported until now including *GLC3A* [7], *GLC3B* [8], *GLC3C* [9] and *GLC3D* [10] at position 2p21, 1p36, 14q24.3 and 14q24.2-q24.3 respectively [10]. Among these loci, mutations in cytochrome P4501B1 (*CYP1B1*) at *GLC3A* and Latent Transforming growth factor-β-binding Protein-2 (*LTBP2*) at *GLC3D* have been reported to cause PCG [11]. However, variants in myocilin (*MYOC)* [12], Forkhead Box C1 (*FOXC1*) [13], and the angiopoietin receptor (*TEK)* [14] have also been reported to be implicated in PCG phenotype.

*CYP1B1* gene has three exons out of which the last 2 codes for a 543 amino acid (a.a) protein [15]. Cytochrome P4501B1 is a heme-thiolate monooxygenases that oxidizes multiple compounds including xenobiotics, steroids, retinoic acid and melatonin [5, 15]. This membrane bound protein has a transmembrane domain at amino terminal (53 a.a) and a highly conserved cytoplasmic region (480 a.a) that is connected to amino terminal by a proline rich hinge (10 a.a) [16]. Exact function of *CYP1B1* in development of eye is uncertain however it is believed that due to mutations in this gene, generation of some important morphogens is affected leading to structural defects in trabecular meshwork and the aqueous humour outflow pathways [5, 6, 17, 18]. Up till now almost 270 mutations are reported in *CYP1B1* gene including missense, small deletions, indels, gross deletions and regulatory mutations [19]. Studies have revealed several genetic mutations causing PCG from Pakistani population, but this data is still too limited as compared to high prevalence of this disorder in our population due to consanguinity [6, 10, 15, 20]. In an ongoing effort of mutation screening of *CYP1B1* gene in PCG cases belonging to consanguineous Pakistani families, we enrolled and screened twenty-five families for *CYP1B1* variants. Each family had at least one child affected with primary congenital glaucoma.

## Materials and methods

### Assessment and enrollment of patients

Based on clinical assessment provided by ophthalmologists, 25 diagnosed families of PCG were enrolled belonging to Khyber Pakhtunkhwa (KPK), Baluchistan and South Punjab. Clinical data, family history and blood samples of patients and each available family member was collected after informed written consent following the principles of world medical association of Helsinki [21]. The study was approved by Bioethical review Committee (BEC) of Quaid-i-Azam University (QAU) Islamabad, Pakistan. Inclusion criteria for patients was diagnosis of PCG through ophthalmologist based on symptoms like buphthalmos, edema and corneal cloudness. Patients with other eye diseases and PCG patients that did not belong to consanguineous couples were excluded from the study. Each family was given a unique identification number (PCG047-PCG069 and PCG101, PCG102). To draw pedigrees of affected families haplopainter program (http://haplopainter.sourceforge.net/about.html) [22] was used.

### Extraction of genomic DNA

Average 4ml of peripheral blood sample was taken from each participating individual and stored in 5ml EDTA (Ethylene Diamine Tetra Acetic acid) vacutainer. Extraction was performed using non-organic method of DNA extraction described by Kaul *et al*., 2010 [23]. To check purity and quantify DNA, Nanodrop was used (Thermo Scientific Nanodrop spectrophotometers).

### Amplification and sequencing of *CYP1B1* gene

Amplification of coding regions and at least 50 base pairs of flanking non-coding regions was performed using primers reported previously by Afzal *et al*., 2019 [20]. 25μl polymerase chain reaction (PCR) was performed for both affected and non-affected individuals following protocol described by Afzal *et al*., 2019 [20]. After amplification, 1.5% agarose gel was prepared to load samples and controls along with DNA ladder (1kb) to separate bands according to their sizes. Purification was done using instructions provided by manufacturer of PCR purification kit (Wiz Bio Solutions, Seongnam, Korea). Finally, each amplified product was sequenced using big dye terminator ready reaction mix (Applied Biosystems, Foster City, CA, USA) in an automated ABI 3100 genetic analyzer. Sequencing results were analyzed by aligning them to reference sequence NM_000104.4 using Sequencher software (5.4.6) and Codon Code Aligner program to identify sequence variations. After identification of each variant, their disease causing potential was checked using Mutation taster (https://www.mutationtaster.org/) [24]. Furthermore, segregation with disease phenotype was confirmed by sequencing other available family members. Each novel identified sequence variant was checked in 96 control samples.

### In silico analysis of variants

For significance of each variant and to check their nomenclature according to Human Genome Variation Society HGVS (http://www.hgvs.org/) guidelines, Mutalyzer (2.0.35) (https://mutalyzer.nl/) [25] was used. Varsome (https://varsome.com/) [26] was used to evaluate the effect of variations and HSF (Human Splicing Finder version 3.1) (https://www.genomnis.com/access-hsf) was used to determine pathogenicity due to disruption of splicing signals because of sequence variants.

### Prediction of variant effect on protein structure and stability

PolyPhen-2 (Polymorphism Phenotyping v2) (http://genetics.bwh.harvard.edu/pph2/) [27], SIFT (Sorting Intolerant From Tolerant) (https://sift.bii.a-star.edu.sg/) [28] and PROVEAN (Protain Variation Effect Analyzer) (http://provean.jcvi.org/seq_submit.php) [29] were used to predict the effect of amino acid substitution on protein structure and function based on sequence homology. I-Mutant v2.0 (https://folding.biofold.org/i-mutant/i-mutant2.0.html) [30] and MUpro (http://mupro.proteomics.ics.uci.edu/) [31] softwares were used which predict protein stability by analyzing thermodynamics based on SVM (support vector method). Both these softwares give Gibbs free energy (ΔΔG = ΔG_*f*_^*wt*^-ΔG_*f*_^*mut*^) of protein structure that corresponds to stabilizing or destabilizing effect of variations. To check the conservation between different mammalian species, Clustal omega (https://www.ebi.ac.uk/Tools/msa/clustalo/) [32] was used and for generation of graphical representation of amino acids WebLogo software (https://weblogo.berkeley.edu/) [33] was used. HOPE software (https://www3.cmbi.umcn.nl/hope/) [22] was used to predict the biochemical changes in structure of protein due to sequence variation.

## Results

**Fig 1:**
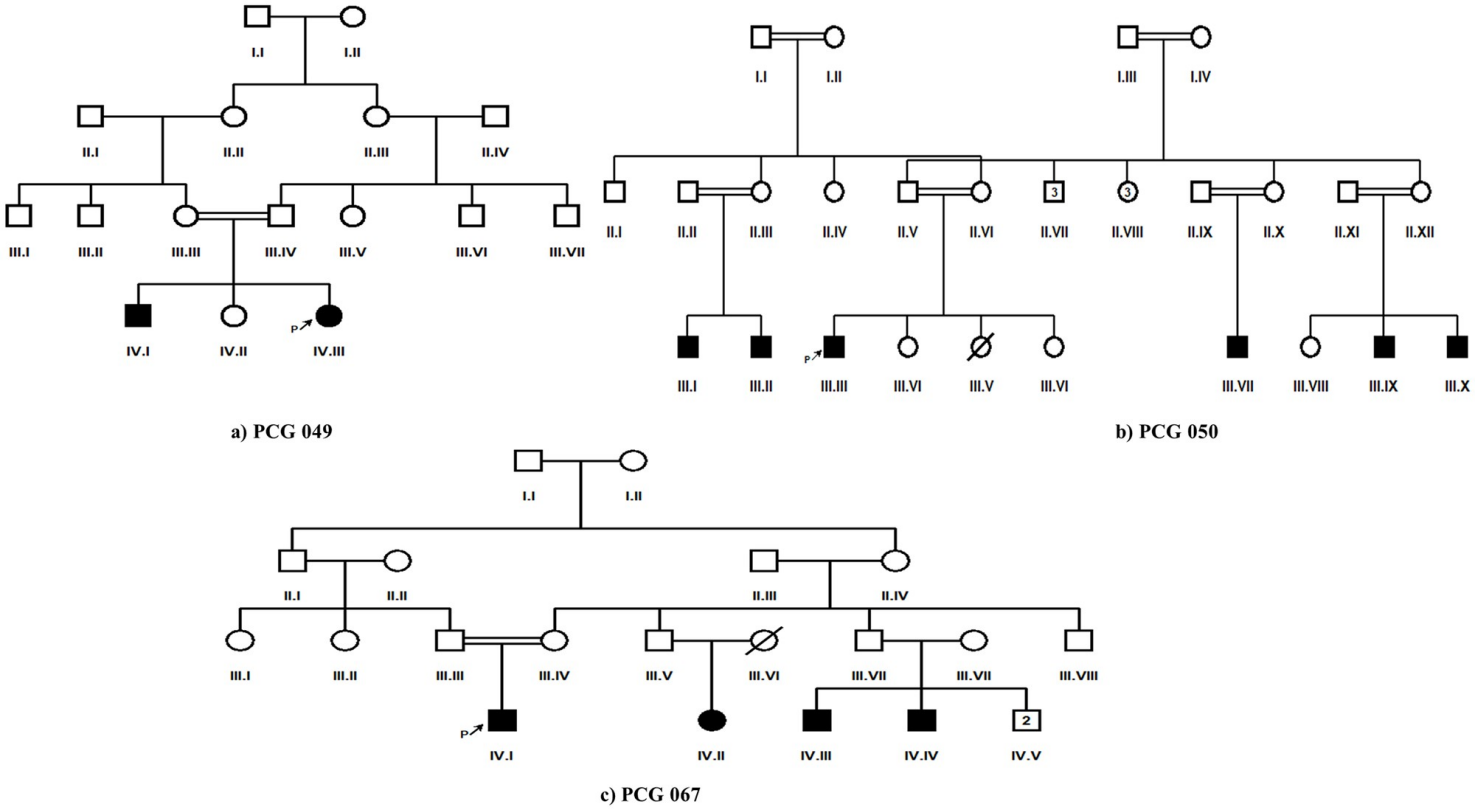
Pedigrees of three PCG families (PCG049, PCG050 and PCG067) having more than one affected member and segregating novel disease causing variant/s in *CYP1B1* gene. Filled squares and circles indicate affected members of family. Cousin marriage is indicated by double line.

In this study, twenty-five PCG segregating consanguineous Pakistani families were enrolled. Among these families i.e., 09 belonged to Punjab province of Pakistan, 01 to Azad Kashmir whereas 12 and 03 belonged to Khyber Pakhtunkhwa and Baluchistan respectively. At the time of enrollment, seventeen families (PCG047, PCG048, PCG052-PCG056, PCG059-PCG063, PCG065-PCG067, PCG101 and PCG102) had a single PCG affected individual, three families (PCG049, PCG057, and PCG065) had two, one family (PCG058) had three, three families (PCG064, PCG051 and PCG067) had four whereas family PCG050 had six affected members respectively (Fig 1).

**Fig 2:**
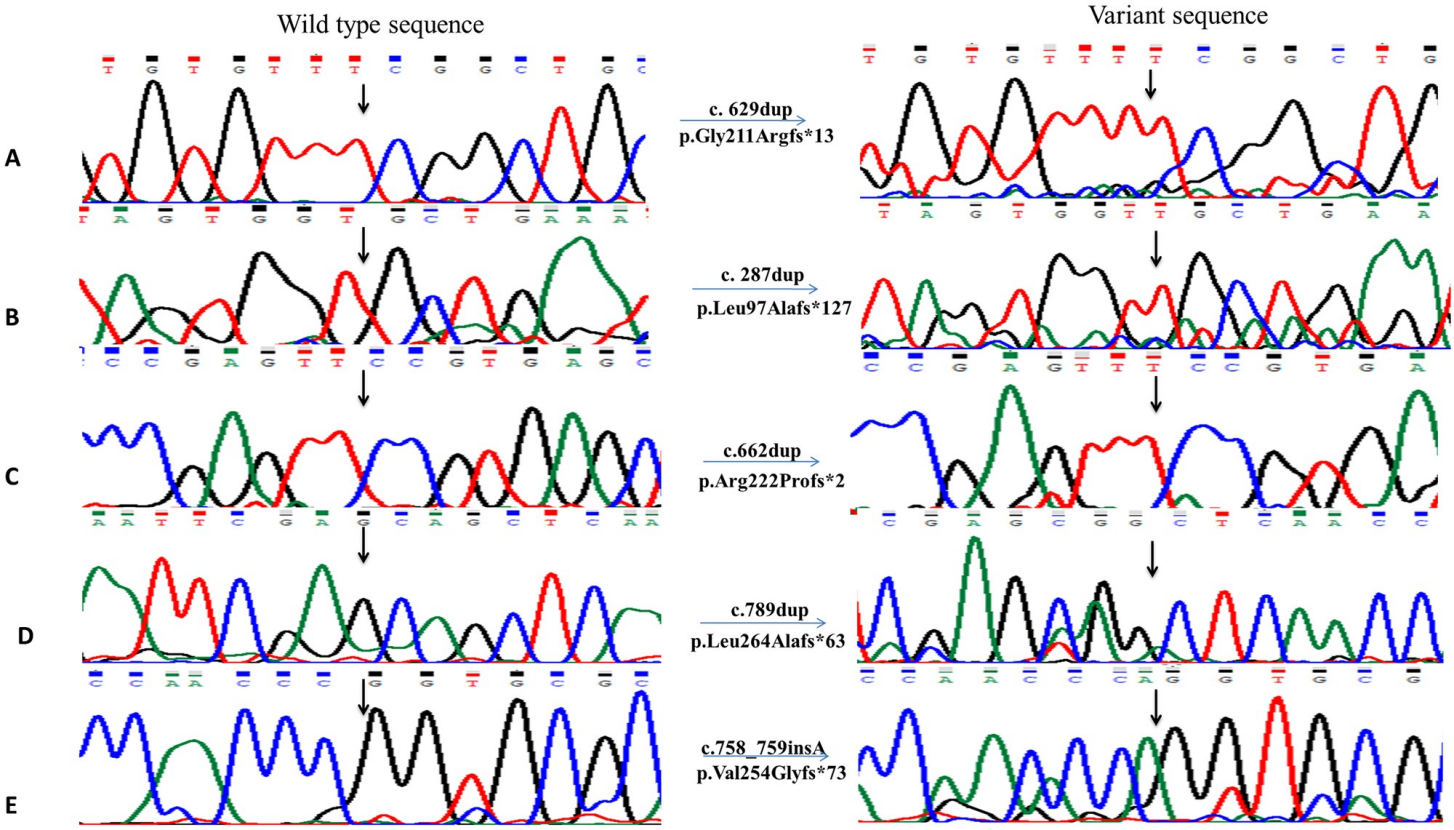
Chromatograms of novel duplications and an insertion detected in PCG patients. **A)** A homozygous variant c.629dup detected in PCG050 leading to p.Gly211Argfs*13. **B)** Chromatogram of homozygous variant c.287dup detected in PCG053 that resulted in protein change i.e., p.Leu97Alafs*127. **C)** Sequence chromatogram of homozygous variant c.662dup leading to protein change p.Arg222Profs*2 in PCG054. **D)** Chromatogram showing c.789dup homozygous variant in PCG063 leading to a p.Leu264Alafs*63. **E)** Homozygous insertion c.758_759insA detected in PCG059 resulting in p.Val254Glyfs*73. All the chromatograms on left side indicate normal sequence while right side of figure shows mutated chromatograms.

**Fig 3:**
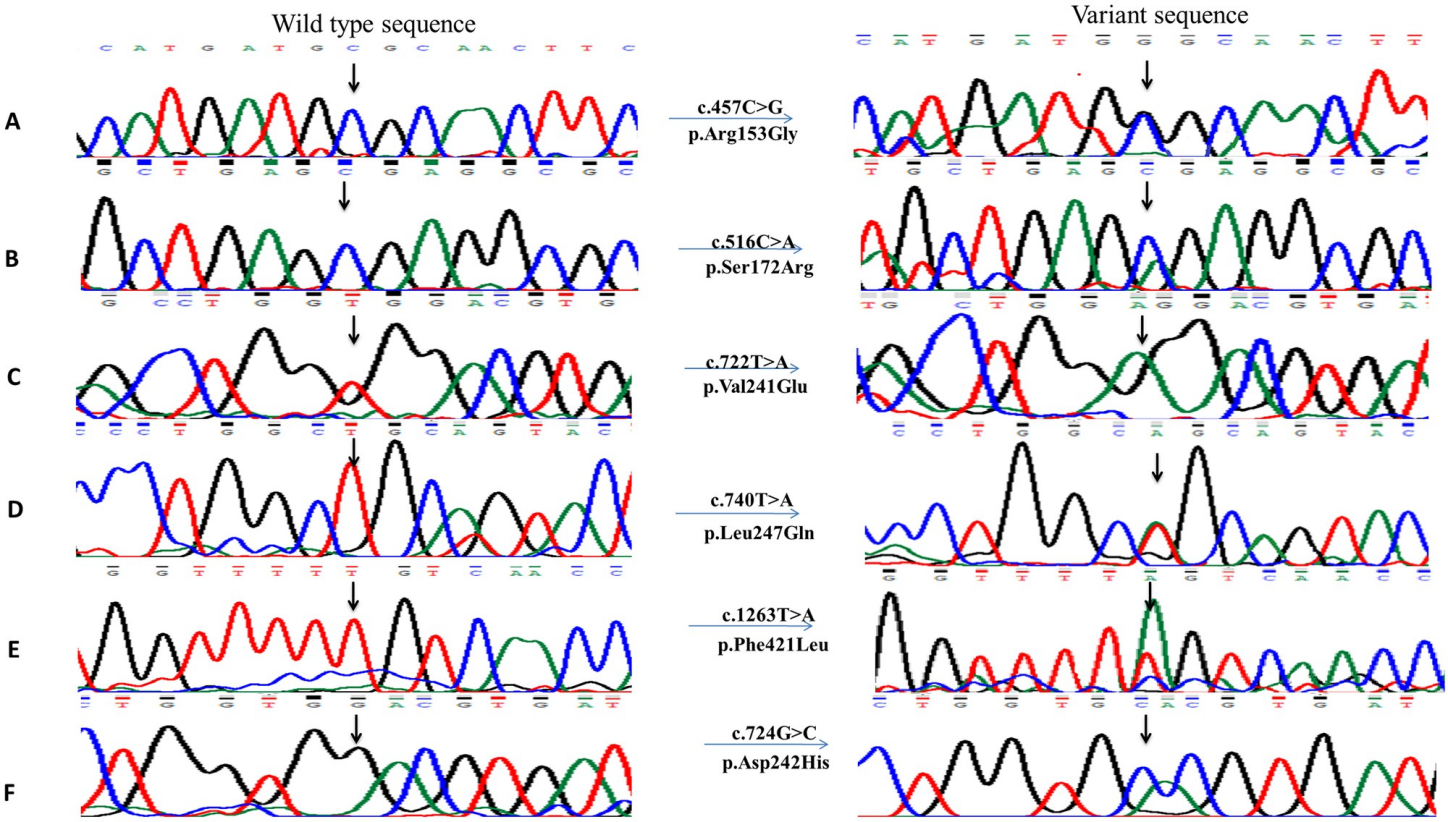
Chromatograms of novel disease-causing single nucleotide substitutions detected in PCG patients upon sequencing of *CYP1B1* gene. Right side of figure shows substituted nucleotides in chromatograms. **A)** A heterozygous variant c.457C>G detected in PCG049 leading to p.Arg153Gly. **B)** Second heterozygous variant c.516C>A detected in PCG049 resulting in p.Ser172Arg. **C)** Variant c.722T>A detected in homozygous condition in PCG052 leading to protein change p.Val241Glu. **D)** A heterozygous variant c.740T>A leading to p.Leu247Gln detected in PCG060. **E)** Sequence chromatogram of heterozygous variant c.1263T>A detected in PCG062 resulting in p.Phe421Leu. **F)** A homozygous variant c.724G>C leading to p.Asp242His detected in PCG067.

Average age of proband of each enrolled family was 10 ± 6 years. Ophthalmological findings confirmed diagnosis of congenital glaucoma for each proband. Proband of each enrolled family had consanguineous mating parents. Molecular screening of *CYP1B1* coding regions and at least 50 base pairs of flanking noncoding region using DNA of each proband revealed thirteen novel disease-causing variations in coding regions according to mutation taster (Table 1, Figs 2 and 3).

**Table 1:** List of reported and novel disease-causing variants detected in this study upon sequencing of *CYP1B1* gene in PCG patients.

Family ID	Position	Nucleotide change	Protein change	Zygosity	Mutation taster prediction	dbSNP Status
**PCG049**	EXON 2	c.457C>G	p.Arg153Gly	Heterozygous	Disease causing	Not reported
	EXON 2	c.516C>A	p.Ser172Arg	Heterozygous	Disease causing	Not reported
**PCG050**	EXON 2	c. 629dup	p.Gly211Argfs*13	Homozygous	Disease causing	Not reported
**PCG052**	EXON 2	c.722T>A	p.Val241Glu	Homozygous	Disease causing	Not reported
	EXON 2	c.732G>A	p.Met244Ile	Homozygous	Disease causing	Reported
**PCG053**	EXON 2	c. 287dup	p.Leu97Alafs*127	Homozygous	Disease causing	Not reported
**PCG054**	EXON 2	c.662dup	p.Arg222Profs*2	Homozygous	Disease causing	Not reported
	EXON 2	c.868dup	p.Arg290Profs*37	Homozygous	Disease causing	rs67543922
**PCG058**	EXON 2	c.247del	p.Asp83Thrfs*12	Homozygous	Disease causing	Reported
**PCG059**	EXON 2	c.758-759insA	p.Val254Glyfs*73	Homozygous	Disease causing	Not reported
**PCG060**	EXON 2	c.740T>A	p.Leu247Gln	Heterozygous	Disease causing	Not reported
**PCG062**	EXON 3	c.1263T>A	p.Phe421Leu	Heterozygous	Disease causing	Not reported
	EXON 3	c.1314G>A	p. (=)	Heterozygous	Disease causing	Not reported
**PCG063**	EXON 2	c.771T>G	p. (=)	Heterozygous	Disease causing	Not reported
	EXON 2	c.789dup	p.Leu264Alafs*63	Homozygous	Disease causing	Not reported
**PCG067**	EXON 2	c.724G>C	p.Asp242His	Homozygous	Disease causing	Not reported

Among these variations seven were found in homozygous state and six in heterozygous state. In addition to novel disease-causing variations three already reported mutations (c.868dup), (c.247del), (c.732G>A) and six reported polymorphisms (c.1347T>C), (c.1294G>C), (c.1358A>G), (c.2244_2245insT), (c.355G>T), (c.142C>G) were also found. Another polymorphism g.35710_35711insT was also found in intronic region in family PCG062 that has not been reported previously. Out of the novel disease-causing variants, five were frame shift variations c.629dup (p.Gly211Argfs*13), c.287dup (p.Leu97Alafs*127), c.662dup (p.Arg222Profs*2), c.758_759insA (p.Val254Glyfs*73) and c.789dup (p.Leu264Alafs*63). Other novel disease-causing variants include six missense variants c.457C>G (p.Arg153Gly), c.516C>A (p.Ser172Arg), c.722T>A (p.Val241Glu), c.740T>A (p. Leu247Gln), c.1263T>A (p.Phe421Leu), and c.724G>C (p.Asp242His) and two silent variations c.1314G>A and c.771T>G. In addition to disease causing variants, seven polymorphisms were also detected (Table 2).

**Table 2:** List of reported and a previously unreported single nucleotide polymorphism detected in *CYP1B1* gene in PCG patients analyzed in this study.

Family ID	Position	Nucleotide change	Zygosity	Protein change	Mutation taster	Polyphen-2	SIFT	Provean	dbSNP
**PCG047-054, 056, 058, 060–064069, 102**	EXON 3	c.1347T>C	Homozygous	p. (=)	Polymorphism	N/A	N/A	N/A	rs1056837
**PCG049, 055, 057**	EXON 3	c.1294G>C	Homozygous	p.Val432Leu	Polymorphism	Benign 0.00	Tolerant 1.00	N/A	rs1056836
**PCG051, 063**	EXON 3	c.1358A>G	Homozygous	p.Asn453Ser	Polymorphism	Possibly damaging 0.906	Tolerant 1.00	Deleterious-3.24	rs1800400
**PCG052, 064, 065, 102**	3’UTR	c.2244_2245insT	Homozygous	p. (=)	Polymorphism	N/A	N/A	N/A	rs4646431
**PCG053, 056, 069**	EXON 2	c.355G>T	Homozygous	p.Ala119Ser	Polymorphism	Benign 0.00	Tolerant 1.00	Neutral 1.51	rs1056827
**PCG056**	EXON 2	c.142C>G	Homozygous	p.Arg48Gly	Polymorphism	Benign 0.00	Tolerant 0.82	Neutral -0.085	rs10012
**PCG062**	Intron	g.35710_35711insT	Homozygous	p. (=)	Polymorphism	N/A	N/A	N/A	Not reported

### *CYP1B1* disease causing variations segregating in families with PCG

In family PCG049, two disease causing variations were detected. A single nucleotide substitution i.e., c.457C>G was present in heterozygous condition, it changed arginine at position 153 to glycine and was deleterious according to PROVEAN and Polyphen-2 with a score of -5.21 and 1.00 respectively (Fig 3A) (Table 3). The other missense heterozygous variant i.e., c.516C>A resulted in substitution of arginine at position 172 (Fig 3B). Human splicing finder predicted that addition of glycine at position 172 will create new sites for auxiliary factors like exonic splicing enhancer (ESE) 9G8, exonic splicing suppresser (ESS) hnRNPA1, IIE, Fas ESS, Sironi_motif2 and break sites for EIE, ESE_SRp55 and Sironi_motif1 that might affect the protein structure.

**Table 3:** In-silico analysis data of disease-causing variants identified in this study.

Nucleotide change	Protein change	Polyphen-2	PROVEAN	Varsome	I mutant		MUpro		HOPE Conservation prediction
					ΔΔG (kcal/mol) DDG value	Stability Prediction SVM2	ΔΔG (kcal/mol)	Stability Prediction SVM2	
c.457C>G	p.Arg153Gly	Probably damaging 1.00	Deleterious -5.21	Likely Pathogenic	-1.47	Decreased Stability	-1.1669	Decreased Stability	Probably stable
c.516C>A	p.Ser172Arg	Possibly damaging 0.685	Neutral -1.167	Pathogenic	-0.03	Decreased Stability	-0.2848	Decreased Stability	Probably stable
c. 629dup	p.Gly211Argfs*13	Probably damaging 1.00	Deleterious -6.66	Pathogenic	-1.26	Decreased Stability	-0.6032	Decreased Stability	Probably stable
c.722T>A	p.Val241Glu	Probably damaging 1.00	Deleterious -4.11	Likely Pathogenic	-0.62	Decreased Stability	-1.5608	Decreased Stability	Damaging
c.732G>A	p.Met244Ile	Benign 0.338	Neutral -0.98	Likely Pathogenic	-0.11	Decreased Stability	-0.5974	Decreased Stability	Probably stable
c. 287dup	p.Leu97Alafs*127	Probably damaging 1.00	Deleterious -4.09	Pathogenic	-0.72	Decreased Stability	-1.9697	Decreased Stability	Probably stable
c.662dup	p.Arg222Profs*2	Probably damaging 0.998	Neutral -2.367	Pathogenic	-0.51	Decreased Stability	-0.8527	Decreased Stability	Damaging
c.868dup	p.Arg290Profs*37	Probably damaging 1.00	Deleterious -6.40	Pathogenic	-1.91	Decreased Stability	-0.9609	Decreased Stability	Damaging
c.247del	p.Asp83Thrfs*12	Possibly damaging 0.732	Deleterious -4.11	Likely Pathogenic	-0.79	Decreased Stability	-1.2898	Decreased Stability	Probably stable
c.758-759insA	p.Val254Glyfs*73	Probably damaging 1.00	Deleterious -3.95	Pathogenic	-3.18	Decreased Stability	-2.691	Decreased Stability	Damaging
c.740T>A	p.Leu247Gln	Probably damaging 1.00	Deleterious -5.45	Likely Pathogenic	-2.14	Decreased Stability	-1.1804	Decreased Stability	Probably stable
c.1263T>A	p.Phe421Leu	Probably damaging 1.00	Deleterious -5.43	Likely Pathogenic	-1.06	Decreased Stability	-0.4212	Decreased Stability	Stable
c.1314G>A	p. (=)	N/A	N/A	Likely Benign	N/A	N/A	N/A	N/A	N/A
c.771T>G	p. (=)	N/A	N/A	Likely Benign	N/A	N/A	N/A	N/A	N/A
c.789dup	p.Leu264Alafs*63	Probably damaging 0.998	Deleterious -3.62	Likely Pathogenic	-1.59	Decreased Stability	-1.5125	Decreased Stability	Deleterious
c.724G>C	p.Asp242His	Probably damaging 1.00	Deleterious -6.50	Likely Pathogenic	-2.35	Decreased Stability	-0.7293	Decreased Stability	Probably stable

*N/A Not available

Insertion of thymine (T) in PCG050 in exon 2 at position 629–630 (Fig 2A) changed amino acid glycine to arginine resulting in frameshift and in-frame stop codon leading to truncated protein after 13 residues (Table 1). This homozygous variant was predicted as damaging for the protein structure according to in-silico analysis (PROVEAN -6.66 and polyphen-2 1.00) (Table 3). Varsome predicted it as pathogenic and negative values determined by I-Mutant and MUpro showed destabilizing effect. HSF analysis for c.629dup predicted alteration of auxiliary sequences i.e., SRp55/SRSF6 (Serine and Arginine Rich Splicing Factor 6) ESE site TTCGGC and Fas ESS site TGTTTC was broken. Two new ESS sites TTTTCG and GTTTTC were created for IIE and one TGTGTTTT for PESS (putative exonic splicing silencer).

In family PCG052, two homozygous variants c.722T>A (Fig 3C) and c.732G>A were found in second exon of *CYP1B1* gene (Table 1). Variation c.732G>A was previously reported and described as less lethal than other variation according to pathogenicity prediction softwares. I-Mutant and MUpro gave negative values for both variations that depicts unstable protein structure (ΔΔG (kcal/mol) -0.62, -0.11 and -1.5608, -0.5974 respectively) (Table 3). In family PCG053, insertion of single nucleotide at position c.287dup (Fig 2B) resulted in a frameshift and in-frame stop codon at 127 position i.e., p.Leu97Alafs*127. This homozygous variant had deleterious effect on protein structure (PROVEAN score -4.09) and was predicted as pathogenic by Varsome (Table 3). This variation was predicted to create ESE/ESS site GGTTGCTG, GTGGTT and TAGTGGTT for factors ESE_SC35, Fas ESS and PESS respectively. Thermodynamics softwares predicted this variation as destabilizing but HOPE described that it might not cause disease because in rare cases this mutant residue was observed in homologous proteins. Two disease causing variants in homozygous condition were present in proband of family PCG054 among which one was already reported i.e., c.868dup that shifted the reading frame and truncated the protein after 37 residues i.e., p.Arg290Profs*37 (Table 1). The other variant was a novel frameshift variation c.662dup (p.Arg222Profs*2) (Fig 2C). Decrease in the stability of protein structure was predicted by I-Mutant and MUpro giving negative energy values -0.51 and -0.852 respectively.

A reported homozygous frameshift variation was found in family PCG058 i.e., c.247del (p.Asp83Thrfs*12) that replaced aspartic acid at position 83 to threonine shifting reading frame and creating stop codon after 12 residues (Table 1). I-Mutant and MUpro gave negative ΔΔG (kcal/mol) values (-0.79, -1.289) for c.247del that corresponds to slightly unstable structure. Insertion of adenine at position c.758-759insA (Fig 2E) resulted in frame shift of 73 bases replacing valine at position 254 to glycine in family PCG059. In-silico analysis described this variant as highly pathogenic (polyphen-2 1.00, PROVEAN -3.95). Highest value (-3.18 kcal/mol) of negative Gibbs free energy was obtained by I-Mutant and by MUpro (-2.691) for this variant (Table 3). Missense variations present in family PCG060 i.e., c.740T>A (p.Leu247Gln) and PCG067 i.e., c.724G>C (p.Asp242His) (Fig 3D and 3F) were predicted to be highly deleterious (PROVEAN score: -5.45 and -6.50) by pathogenicity prediction tools. Change of aspartate at position 242 to histidine resulted in creation and destruction of many sites for auxiliary factors that help in splicing. Sites for ESE_9G8, EIE, Sironi_motif2, PESE, Sironi_motif1 were broken and new site was created for ESE_SRp55CACGTG according to HSF.

Family PCG062 had two different heterozygous variants including c.1263T>A (Fig 3E) and c.1314G>A in coding regions (Table 1) showing compound heterozygosity. One of the detected variant c.1263T>A changed phenylalanine at position 421 to leucine and was reported as probably damaging by Polyphen-2. Human splicing finder predicted that a new acceptor site will be created CTGTGGTTTTTGTC>CTGTGGTTTTAGTC changing consensus value (CV) from 50.91 to 78.78. CV for newly created site showed that it is not a very strong site (strong site CV> 80). PCG063 had two unreported mutations, a silent heterozygous mutation c.771T>G and a frameshift homozygous mutation c.789dup (Fig 2D). Shifting the frame by 63 amino acids replaced leucine at position 264 to alanine and resulted in a short protein (Table 3). HSF analysis showed that CAGGCT site was created for ESS_hnRNPA1, CAGGCTCA for PESE, GCAGGC for ESE_9G8 and AGCAGC, AGCAGCTC sites were broken that are required for auxiliary factors ESE_SRp55 and PESE respectively.

**Fig 4:**
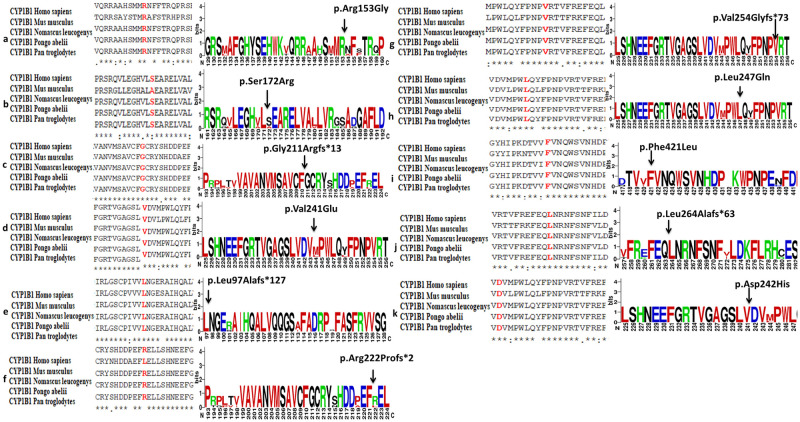
A comparison of *CYP1B1* gene conservation among different homologs for novel variants detected in this study. **a)** Clustal Omega multiple sequence alignment (MSA) (Shown in red) for p.Arg153Gly detected in PCG049. **b)** Multiple sequence alignment (MSA) for p. Ser172Arg among homologs detected in PCG049 showing less conservation in *Mus musculus*. **c)** MSA result for p.Gly211Argfs*13 among homologs detected in PCG050. **d)** MSA result for variant p.Val241Glu detected in PCG052. **e)** MSA result for p.Leu97Ala*127 among homologs detected in PCG053. **f)** MSA result for p.Arg222Profs*2 detected in PCG054 showing less conservation in *Mus musculus*. **g)** MSA result for variant p.Val254Glyfs*73 detected on PCG059 showing complete conservation among homologs. **h)** MSA result for p. Leu247Gln among homologs detected in PCG060. **i)** MSA result for p.Phe421Leu detected in PCG062. **j)** MSA result for p.Leu264Alafs*63 detected in PCG063. **k)** MSA result for p.Asp242His detected in PCG067. **a-k)** WebLogo results of all novel CYP1B1 protein variants showing comparison of conservation among homologs are on right side of the figure. All the variants except p.Ser172Arg and p.Arg222Profs*73 show 100% conservation among different mammals (Large size of amino acid abbreviation letter show full conservation while small size show less conserved position among homologs).

**Fig 5:**
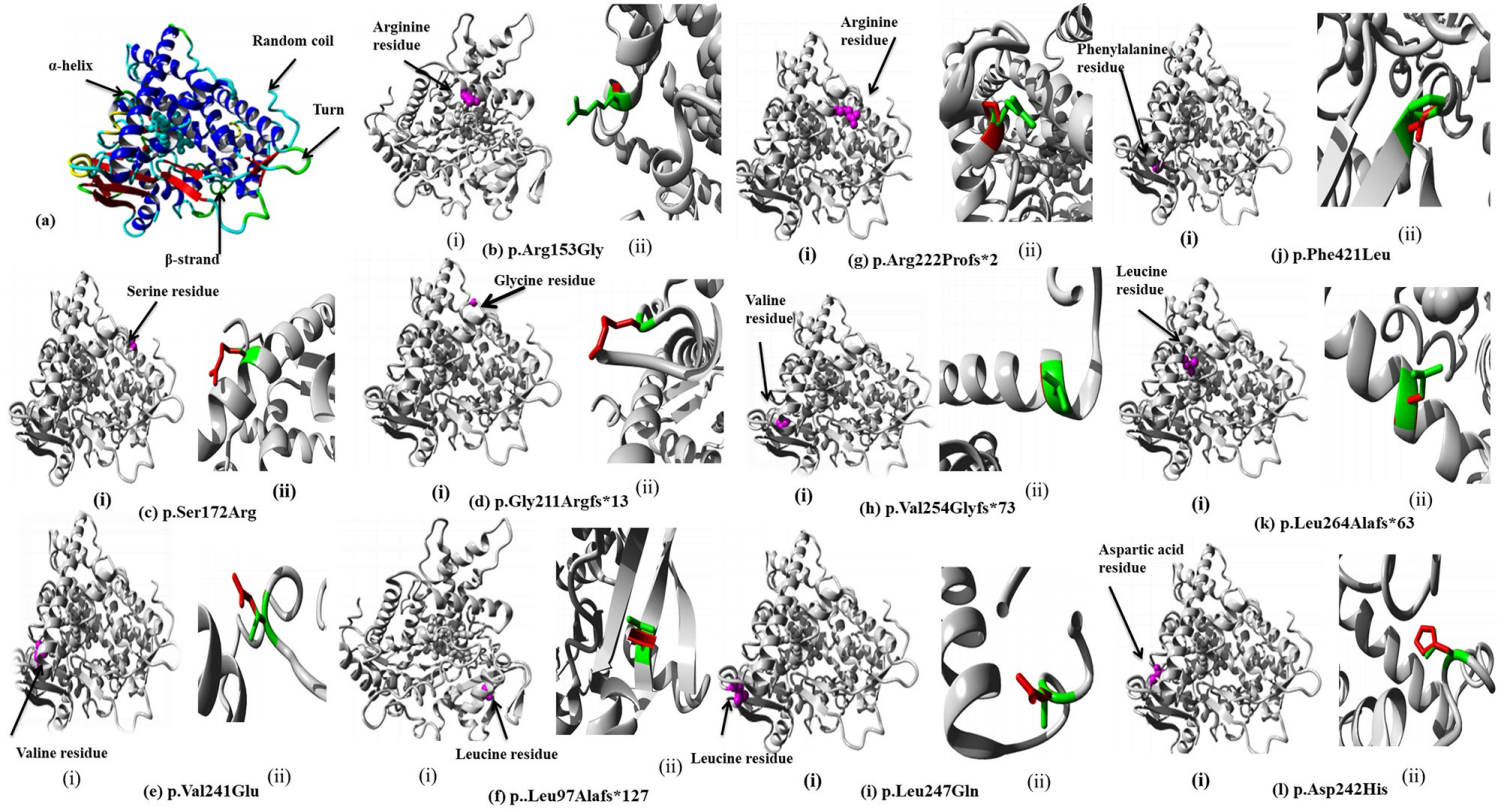
(a) Overview of CYP1B1 wild type protein in ribbon-presentation (b-l) In-silico protein prediction regarding variant protein structures for all detected variants by HOPE software. (b-l) i: for each variant show the position of amino acid in protein structure. (b-l) ii: for each detected variant show the zoomed in change in protein structure due to each respective variant (Wild type amino acid residue is colored as green while mutant is colored as red).

Amino acid conservation was analyzed by using Clustal Omega multiple sequence alignment tool among CYP1B1 homologous sequences from different species i.e.: *Mus musculus* (NP_001075448.1), *Nomascus leucogenys* (XP_003262792.2), *Pongo abelii* (XP_009235654.1) and *Pan troglodytes* (XP_001167556.1) with high similarity index to *Homo sapiens* (Fig 4a–4k). Structures predicted by HOPE software showed the position of mutant residues and their possible impact on conservation of protein structure (Fig 5a–5l) (Table 3).

### CYP1B1 polymorphisms in families with PCG

A single nucleotide polymorphism, c.1347T>C (rs1056837) (Table 2) was found in homozygous state in highest frequency 44% in enrolled families (PCG047, 054, 056, 058, 060, 061, 062, 063, 064, 069 and PCG102). Second variation c.1294G>C (rs1056836) (Table 2) was found in PCG049, 055 and 057 (12%) also showed amino acid change p.Val432Leu that was described as Benign 0.00 by PolyPhen-2. Family PCG051 and 063 (8%) showed SNP c.1358A>G (rs1800400) (Table 2) in coding region that changed amino acid at position 453 from asparagine to serine. This polymorphism was predicted as possibly damaging with a score of 0.906 by PolyPhen-2 and deleterious with a score of -3.24 by PROVEAN. Insertion of T (c.2244_2245insT) (rs4646431) (Table 2) was found in family PCG052, 064, 065 and 102 (16%) in 3’untranslated region (3’UTR). In exon 2 a substitution of G by T (c.355G>T) (rs1056827) was detected in proband of family PCG053, 056, 069 (12%) (Table 2) in homozygous condition leading to protein change i.e., p.Ala119Ser with no pathogenic effect as predicted by in-silico analysis. Family PCG056 showed SNPs c.142C>G (rs10012) in addition to most prevalent variation c.1347T>C in coding region. This polymorphism was considered as benign and neutral by pathogenicity prediction tools. In this variation arginine at position 48 is replaced by glycine (p.Arg48Gly). Family PCG062 also revealed a previously unreported polymorphism g.35710_35711insT (Table 2) in intronic region.

## Discussion

High frequency i.e., 70–100% of consanguineous marriages [34] is the main cause of high prevalence of autosomal recessive disorders like PCG in Pakistan [6]. Mutated *CYP1B1* coded protein is reported to cause abnormal development of ocular structures resulting in impeded outflow of aqueous humor and PCG phenotype [35, 36]. Data retrieved through studies have shown that mutational spectrum of in *CYP1B1* gene varies among different populations i.e.; p.Ser476Pro is 44% prevalent in India, p.Arg469Trp, p.Arg368His, p.Arg390His, p.Gly61Glu and p.Glu173Arg are 70% prevalent in Iran, p.Gly61Glu, p. Arg390His and p.Glu229Lys are 80–100% prevalent in Saudi Arabia however p.Arg330Phe and p.Arg390His are predominantly reported from China [37]. Founder mutations reported from India c.1449G>A (R368H), Iran c.182G > A (p.Gly61Glu), Europe c.7996G > A (p.Glu387Lys), Saudi Arabia c.182G > A (p.Gly61Glu) and South Korea c.958G > T (p.Val320Leu) are not prevalent in Pakistan [15]. Previous studies from Pakistan had reported p.Arg390His mutation to be implicated in more than 50% of analyzed PCG cases [6, 20, 38, 39]; however, in present study, we did not identify this mutation in any of the analyzed case. A possible explanation of non-detection of p.Arg390His mutation in our study cohort could be the differences in ethnicities of analyzed subjects. In previous studies, PCG cases belonging to Punjab and Sindh provinces of Pakistan were included [6, 38] however in present study majority of the families i.e., 13/25 belonged to Baluchistan province of Pakistan.

In present study *CYP1B1* analysis in 25 cases enrolled through various regions of Punjab, Baluchistan and Khyber Pakhtunkhwa, Pakistan revealed a total of seven frameshift, seven missense and two silent disease-causing variations. Among seven frameshift variations five are novel however two are previously reported in patients of different ethnicities. The variant c.868dup (p.Arg290Profs*37) (rs67543922) was initially identified in PCG affected Pakistani family by Sheikh *et al*., 2014 [40] and then in another family by Micheal *et al*., 2015 [39]. Mutational analysis of *CYP1B1* conducted on population of Sindh and Punjab province of Pakistan by Rashid *et al*., 2019 [38] reported that out of total 427 individuals, c.868dup was found in two families that resulted in premature stop codon and eventually truncation.

Second reported frame shift variant c.247del (p.Asp83Thrfs*12) was initially identified in a study conducted on Indian population by Tanwar *et al*., 2009 [41]. According to the study a stop codon TAG was introduced at position 94 due to frameshift after codon 82 [41]. Five homozygous frameshift variants including c.629dup, c.287dup, c.662dup, c.4_5insT, c.758_759insA and c.789dup detected in our study are not reported earlier from Pakistan or any other region. All homozygous variants identified in this study showed a perfect segregation with phenotype of disease in all families (Fig 2). Previously, Ou *et al*., 2018 [42] have shown that the active site residues of CYP1B1 are distributed from amino acid 126 to 510 of the protein therefore all truncations that omit one or more of these amino acids result in loss of protein function [43].

Missense disease-causing variants found in family PCG049, 052, 060, 062 and 067 and two silent disease-causing variants found in family PCG062, 063 are also previously unreported (Fig 3, Table 3). In CYP1B1 protein, novel missense variant p.Arg153Gly is located in C-helix, p.Ser172Arg in D-helix, p.Phe421Leu in K-helix, p.Val241Glu, p.Asp242His and p.Leu247Glu in substrate recognition site 2 [42]. The locations of residue replacements in conserved core structures highlight their possible severe affect on mutated protein structure and functionality hence causing disease phenotype [43, 44]. Here in two families PCG049 and PCG062, we identified compound heterozygous mutations in *CYP1B1* gene. Previously compound heterozygosity has been reported in developmental glaucoma, [45] and primary congenital glaucoma patients from China [46]. Cai *et al*., 2021 reported that two heterozygous mutations c.1310C>T (p.P437L) and c.3G>A (p.M1I) are responsible for glaucoma in a Chinese family [45]. In another study conducted on 13 Chinese PCG patients, two heterozygous mutations Ala330Phe and Arg390His were detected in a patient and reduced enzymatic activity due to these variants was reported to be the cause of disease [46]. Waryah *et al*., 2019 identified compound heterozygosity (p.Val364Met along with p.Pro350Thr) in two consanguineous families of PCG belonging to different ethnic groups of Pakistan [47]. Furthermore, previous studies have also reported co-segregation of heterozygous variants of *CYP1B1* with heterozygous *TEK* alleles in PCG cases [48]. In present study, we identified a heterozygous variant p.Leu247Gln in a consanguineous family i.e., PCG060 and absence of any other heterozygous/homozygous variant in *CYP1B1*, recessive inheritance pattern and previously reported allelic interactions of two un linked genes for PCG phenotype [12, 13, 48] necessitates genetic analysis of other glaucoma related genes including MYOC, FOXC1 and TEK genes in PCG060 family.

Due to epigenetic modifications and different environmental factors incomplete penetrance and increased variability could be observed in manifestation of *CYP1B1* disease causing variations in PCG patients [38, 49]. We could not identify homozygous or compound heterozygous disease-causing variants in fifteen analyzed families in this study that predicts the contribution of other genes like *LTBP2*, *TEK*, *MYOC*, *FOXC1* and regulatory effect of cis-acting elements, splicing elements or possible modifiers [12, 13, 40, 50].

All single nucleotide polymorphisms (SNPs) identified in current study except one present in intronic region i.e., g.35710_35711insT in homozygous state are previously reported. Four reported SNPs i.e., rs1056836 (c.1294G>C), rs1800400 (c.1358A>G), rs1056827 (c.355G>T) and rs10012 (c.142C>G) showed amino acid change while two polymorphisms rs1056837 (c.1347T>C) and rs4646431 (c.2244_2245insT) were silent. Most prevalent polymorphism (45%) c.1347T>C in present study was also reported in other studies conducted on PCG cases from Pakistani population [51]. Afzal *et al*., 2019 [20] reported SNP c.142C>G in 23.6%, c.1294G>C in 25.3% and c.355G>T in 53.2% cases while in current data they showed a frequency of 4.1%, 12.5% and 12.5% respectively.

## Conclusion

In conclusion we identified thirteen previously unreported and three reported mutations as well as six SNPs (one novel) in PCG probands born to parents having consanguineous marriages highlighting the autosomal recessive pattern of disease. Proper genetic testing and counseling should be provided to people in high consanguinity areas to help ophthalmologists in disease management and treatment. Mass screening and additional studies are required to better understand the heterogeneous pattern and contribution of *CYP1B1* gene to PCG pathophysiology in our population.
